# Advances in extrusion-based bioprinting enabled by advanced printhead and nozzle designs

**DOI:** 10.1016/j.mtbio.2026.102941

**Published:** 2026-02-16

**Authors:** Jianfeng Li, Peer Fischer

**Affiliations:** a Max Planck Institute for Medical Research, Jahnstr. 29, Heidelberg, 69120, Germany; b Institute for Molecular Systems Engineering and Advanced Materials, Heidelberg University, Im Neuenheimer Feld 225, Heidelberg, 69120, Germany; c Center for Nanomedicine, Institute for Basic Science (IBS), Seoul, 03722, Republic of Korea; d Department of Nano Biomedical Engineering (NanoBME), Advanced Science Institute, Yonsei University, Seoul, 03722, Republic of Korea

**Keywords:** 3D bio-printing, Extrusion printing, Printhead, Nozzle, Tissue engineering, Acoustic field, Magnetic field, Multi-material printing

## Abstract

3D printing is a rapidly evolving technology that enables new applications in biomedical engineering. In particular, its role in the fabrication of complex living tissues and multimaterial structures that support living cells opens new possibilities for biomaterial processing as well as potential clinical applications. Among the various 3D printing modalities developed over recent decades, extrusion-based printing shows particular promise for bioprinting and a number of successful examples are highlighted in this review. However, despite its widespread adoption, extrusion-based 3D printing is constrained by the limited range of viscoelasticities that can be processed, certain inefficiencies in multi-material printing, restricted spatial resolution and fundamental trade-offs between printing speed and cell viability in bioprinting applications. Here, we present a comprehensive review of existing printhead designs for extrusion-based 3D printing, with a specific focus on biomedical applications. We highlight recent technological breakthroughs, identify persistent bottlenecks and propose strategic directions for next-generation printhead development aimed at overcoming current limitations. Our goal is to catalyze innovation in printhead engineering for biomedical applications to enable the fabrication of structures that are still unattainable with current extrusion-based 3D printing systems.

## Graphical abstract

**Figure:**
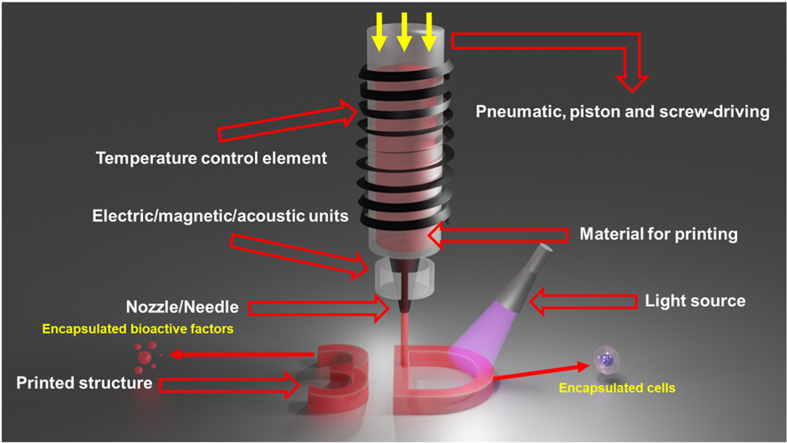

## 1 Introduction

3D printing has emerged as a transformative platform technology for rapid prototyping and regenerative medicine [1,2]. By enabling the direct fabrication of pre-designed digital models or patient-specific geometries derived from 3D imaging data, 3D printing offers customizability, process efficiency and reproducibility. In contrast to conventional formative or subtractive fabrication techniques (e.g., molding, die casting, milling and cutting), 3D printing builds objects additively through spatially controlled deposition of bio- and support-materials.

A broad spectrum of 3D printing technologies has been developed, with many initially conceived for applications beyond bioprinting. These include extrusion-based printing [e.g., direct ink writing [3] and fused deposition modeling (FDM)] [4], ink-jet printing [5], selective laser melting (SLM) [6] and vat-photopolymerization-based printing [e.g., digital light processing (DLP), stereolithography (SLA), continuous light interface production (CLIP) and two-photon polymerization (TPP)] [7]. Each technique offers unique advantages and limitations, lending itself to specific application domains rather than serving as a one-size-fits-all solution, as has also been highlighted in prior comprehensive reviews [[7], [8], [9]]. Among these printing methods, extrusion-based printing has gained significant traction due to its broad material compatibility and operational simplicity [[10], [11], [12]]. The affordability of desktop extrusion-based 3D printers over the past one decade has further increased the access, fostering widespread adoption across academic and industrial research settings. Central to the versatility of extrusion-based systems is the printhead, whose design critically governs parameters such as resolution, throughput and material handling capabilities. Innovations in printhead design have enabled the integration of complex functionalities, such as multi-material co-printing, in situ mixing, gradient structure generation and geometry-guided deposition, pushing the boundaries of achievable structural and material complexity. This also benefits 3D printing for tissue and organ fabrication, which typically involves multiple cell types and intricate internal architectures. Moreover, specific printhead designs can reduce damage or stress to living cells during printing and enable greater control over the organization of printed cellular structures. At present, extrusion-based printing with a single nozzle is the most widely used technology for 3D bioprinting [13,14]. A variety of cell types have been 3D printed using extrusion-based 3D printers, including different types of stem cells [15] and specific cell types derived from a number of tissues [1]. Cell densities used in extrusion-based 3D bioprinting typically range from 0.1 to 10 million cells per milliliter [16]. Higher cell densities present challenges both in formulating 3D printable bioinks prior to printing and in ensuring sufficient nutrient and oxygen diffusion throughout the 3D printed structures after printing. Due to the rheological limitations of bioinks and the shear stress experienced by cells as they pass through the nozzle, the printed feature size is typically larger than 100 μm. However, the resolution can be reduced to approximately 20 μm, which is also roughly the size of a single cell, when printing within suspended hydrogels, which helps maintain the deposited structure [17]. Higher resolution does not necessarily indicate better performance in 3D bioprinting, as it increases printing time and exposes cells to greater shear stress in smaller nozzles. Ideally, printing should be completed as quickly as possible to minimize the stresses imposed on cells during ink preparation, extrusion and structure solidification, and to transfer the printed cell containing constructs into the cell culture incubator promptly or to close the wound if the printing is performed in situ during surgery [18].

In this review, we present a comprehensive overview of recent advances in printhead engineering for extrusion-based 3D printing aimed at enabling biomedical applications. Due to the delicate and complex nature of bio-inks, printhead innovation is particularly critical in this context. Specifically, we examine how emerging designs and functionalities have expanded the technological landscape by overcoming limitations in material processability. By systematically summarizing the advantages and limitations of different printheads, comparing various printhead designs, and analyzing the interplay between bioink formulation and printhead architecture, we aim to provide practical guidance for future research.

## 2 Conventional 3D printheads

**Fig. 1:**
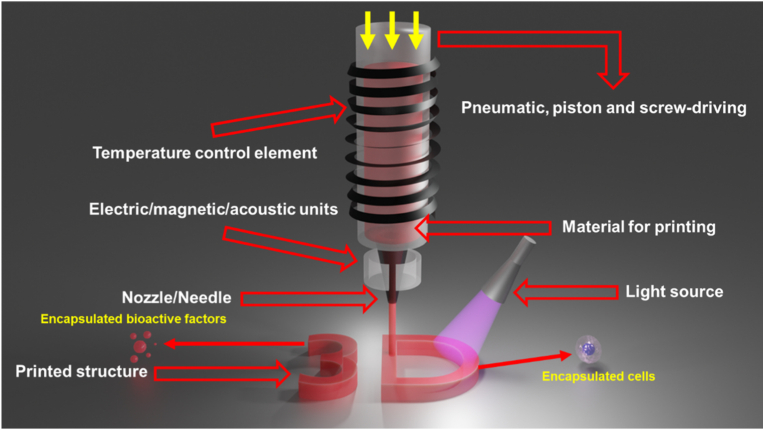
**A typical design of extrusion-based printhead.** Pneumatic, piston and screw-driven mechanisms represent the three predominant dispensing strategies in extrusion-based 3D printing. A temperature control element is often incorporated to regulate the rheological properties of the printing materials, facilitating controlled flow and deposition. The nozzle or needle plays a critical role in determining the printing resolution. Additionally, a light source may be integrated to crosslink light-polymerizable materials, thereby preserving the structural integrity of the printed construct. Electric, magnetic or acoustic units, as discussed in the main text, can be incorporated around the nozzle/needle to assist with shaping the material extrusion and the manipulation of embedded materials within the ink. Living cells and bioactive factors can be encapsulated inside the 3D printed structure.

The printhead serves as the central component of extrusion-based 3D printing, governing the behavior of 3D-printable materials (such as inks, thermoplastic powders, pellets or filaments) prior to deposition. Typically, it comprises a material delivery mechanism, a temperature control module and a dispensing unit, such as a nozzle or needle (Fig. 1).

In extrusion-based 3D printing systems, three primary material delivery mechanisms are employed to drive material extrusion within the printhead: pneumatic, piston and screw-driven systems [19]. Among these, pneumatic systems are the most commonly employed due to their operational simplicity and minimal maintenance requirements. Sterility is maintained more easily within a cartridge when there are no additional parts inside, especially in the case of delicate designs that are especially difficult to clean. In contrast, piston- and screw-driven systems can exert significantly higher mechanical forces, making them better-suited for processing highly concentrated or viscous materials. In particular, screw-driven systems have attracted increasing attention for their superior mixing capabilities and adaptability to a broad range of material systems [20].

There are rheological constraints on the materials (inks) used in extrusion-based 3D printing. Inks should exhibit shear-thinning behavior to enable smooth extrusion through the nozzle and rapidly recover to a solid-like state to maintain the printed structure and support subsequent layers, with the storage modulus (G′) exceeding the loss modulus (G″) in the low-shear regime [21]. Two key rheological parameters need to be considered during ink preparation. One is the yield stress, defined as the minimum stress required to initiate flow, which serves as a critical parameter for 3D printing, particularly in 3D bioprinting. A bioink with an appropriate yield stress is not only essential for achieving high shape fidelity and structural stability in printed constructs, but also crucial for maintaining a uniform cell distribution within the bioink [22]. Another key parameter is the recovery time, defined as the time required for the bioink to restore its solid-like state after the removal of shear stress. A recovery time of 5 to10 s is generally recommended to ensure uniform cell distribution within the bioink and high shape fidelity after printing [23]. As comprehensive review articles on ink design for extrusion-based 3D printing are already available [21,24,25], this topic is not discussed in further detail in this section.

The rheological properties of the feed materials within the printhead can in some cases be precisely modulated through temperature control, which is essential for achieving consistent extrusion and high-resolution print fidelity. Commercial systems often support a broad thermal range (from <0 °C to over 200 °C), thereby allowing precise tuning of rheological behavior to accommodate diverse material formulations. However, especially in bioprinting it is generally not possible to operate at elevated temperatures.

Beyond a temperature control module, additional functional modules can be integrated into the printhead or deposition platform to facilitate in situ post-processing. For example, the integration of an LED light source enables the immediate crosslinking of photo-polymerizable materials during or after deposition, thereby maintaining the structural fidelity of the printed construct (Fig. 1). As we discuss below, extensions that provide magnetic, electric and acoustic fields have been developed, where acoustic fields show particular promise when extruding cell-laden bioinks.

The resolution of printed constructs is largely governed by the material's rheological properties and the diameter of the dispensing orifice. Dispensing components in extrusion-based 3D printing typically utilize standard Luer-lock needles or nozzles. Commercial nozzles are available with internal diameters as small as 50 μm. However, reducing the size of the nozzle necessitates lower-viscosity bioinks with smaller particle sizes and higher driving pressures, which in turn increase the risk of clogging. These constraints are particularly critical in bioprinting, where elevated shear stresses can compromise cell viability [26]. Nevertheless, researchers have attempted to employ tapered capillaries as nozzles to overcome the limitations of conventional nozzle fabrication strategies. Using these tapered capillary-based nozzles, 3D-printed structures can approach feature sizes in the hundreds of nanometers [27,28]. As mentioned, formulated inks with well-tuned viscosities are required and the printing speed is limited because these setups are generally driven by capillary forces rather than applied external pressures. Beyond nozzle dimensions, printing speed also influences fidelity: the motion of the printhead imposes forces on the extruded filament, which can increase the resolution when the filament is stretched [27]. However, excessive printing speeds may cause extruded strand breakage, whereas slower speeds can yield uneven deposition. Moreover, fabricating large constructs at high resolution substantially prolongs printing time, potentially impairing cell viability and biomolecular activity. Thus, careful optimization of nozzle geometry, material formulation and printing parameters is essential to balance resolution, throughput and biological performance in extrusion-based bioprinting.

FDM is the most popular modular extrusion-based 3D printing technology focusing on 3D printing with thermoplastic materials as continuous filaments. For FDM based 3D printing, the printhead incorporates a heating module to melt the filament, which is then extruded through the nozzle and solidifies upon deposition onto the substrate due to rapid temperature dropping. High-resolution FDM printing depends critically on precise temperature control and nozzle orifice dimensions. Researchers have found that nozzle shape has profound influence on the flow profile passing though and optimizing the shape design can improve the overall printing qualities [29]. Nevertheless, FDM is inherently limited to filament-based materials, especially rigid filament, which constrains its material and application versatility. For filament prepared from materials with less mechanical strength, buckling will happen after the filament going through the rollers and preventing from entering the heat element and nozzle. Structures printed using FDM printers have been applied in tissue engineering for metal particle enforced biopolymer scaffold fabrication [30] and personalized medicine to incorporate multiple pharmaceutically-active compounds with different release patterns [31]. However, living cells have not yet been printed with this technique because the printing material must first be prepared in a filament form suitable for feeding. In spite of that, researchers have developed cost-effective methods to convert FDM printers to 3D bioprinters by integrating pneumatic extrusion systems [32,33], thereby further expanding the application of FDM printers in biomedical fields.

## 3 Functional 3D printheads

A typical 3D bioprinting process involves incorporating cells or bioactive factors into a biomaterial matrix to form a printable bioink (Fig. 1). To achieve high print fidelity, the bioink must exhibit shear-thinning behavior and a storage modulus (G′) higher than the loss modulus (G″) in the low-shear regime during extrusion. However, achieving uniformly mixing of cells or bioactive factors within such viscoelastic biomaterials at room temperature while maintaining sterility remains challenging. Strict aseptic handling throughout the preparation is indispensable, yet the process must also be completed rapidly to preserve cell viability and bioactive factors activity. In practical applications, bioinks with varying cell densities or biomaterial compositions are often required, further complicating pre-printing preparation.

As demonstrated in the previous section, conventional 3D printing systems are inherently limited by their inability to spatially control the composition and properties of materials during deposition. Standard printheads are typically designed to extrude a single material in a continuous and uniform way. While this setup is sufficient for basic structural printing or single-phase material fabrication, it presents significant challenges for the fabrication of heterogeneous or functionally graded constructs, as is of interest when printing tissues, that require precise control over the distribution of active components (e.g., cells, bioactive factors), the extruded strand shape and the microscale material composition.

This limitation poses a major challenge in biofabrication, where it is essential to integrate diverse biomaterials, each exhibiting distinct mechanical, physicochemical or biological properties. For instance, in tissue engineering, printing a scaffold that reproduces the native counterpart with a continuous gradient of biomaterial compositions or cell-type distributions is not feasible with a standard printhead. Furthermore, 3D printing on complex substrates is often required in clinical settings, which is likewise incompatible with a standard printhead. For large organ printing, the controlled deposition of bioactive factors and cell types within a well-defined 3D architecture demands far greater control over material processing than conventional printheads can provide. To overcome these limitations, a variety of functional printheads have been developed.

### 3.1 Functional screw driven printhead

**Fig. 2:**
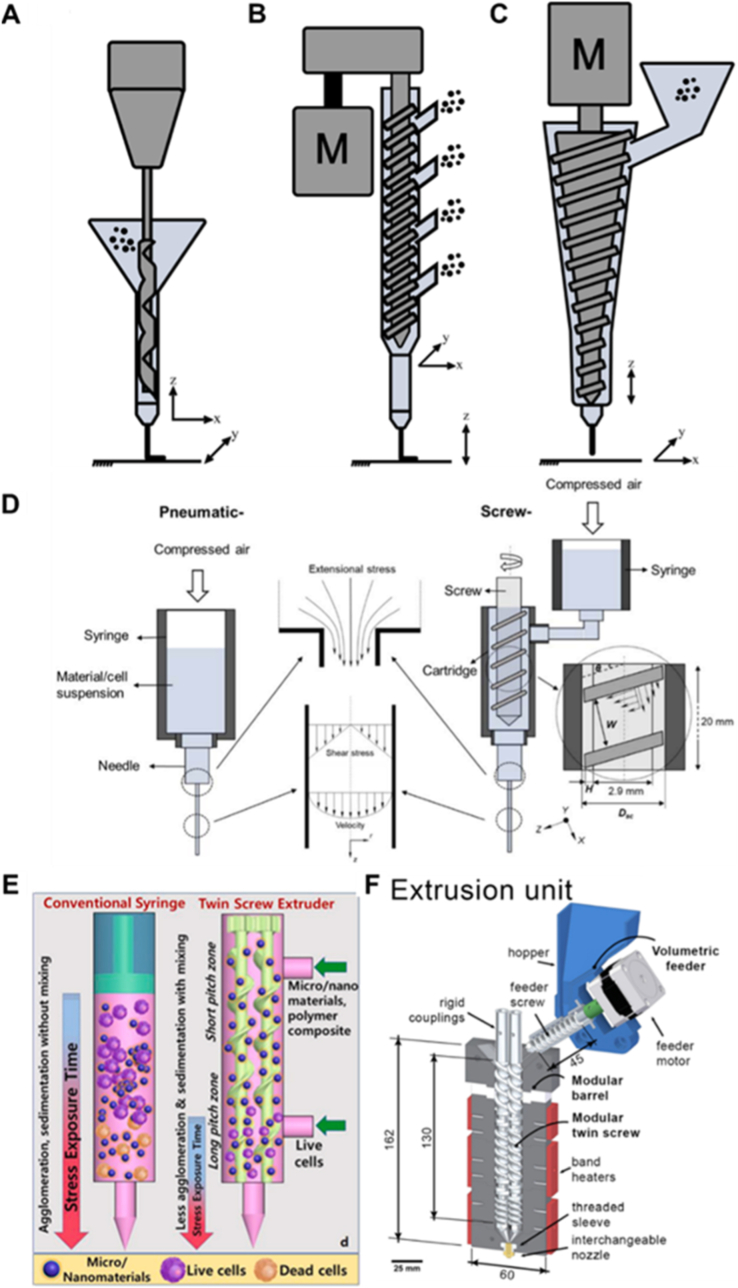
**Some representative designs of functional screw driven printheads.** (A) A CNC system based screw-driven printhead with a drill bit as the extrusion screw [35]. (B) A screw-driven printhead with multiple feeding ports [36]. (C) A screw-driven printhead with a conical screw geometry suitable for highly elastic and flexible materials printing [34,37] (Fig. 2A–C have been adapted with permission from Ref. [34]. Copyright Springer Nature 2021). (D) Cells experience higher stress in the screw-driven printheads than in the pneumatic printhead [38] (Adapted with permission from Ref. [38]. Copyright IOP Publishing Ltd 2020). (E) A screw-driven printhead with different pitch length and two inlet ports for cell containing ink printing [39] (Adapted with permission from Ref. [39]. Copyright Elsevier 2021) (F) A modular dual screw-driven printhead design with improved 3D printing performance [20] (Adapted with permission from Ref. [20]. Copyright Elsevier 2022).

The mixing of bioinks is crucial for reproducible 3D bioprinting. Cell-laden inks should contain homogeneously distributed cells and bioactive factors. Currently, mixing is largely performed manually, which may result in batch-to-batch variation. In addition, bioinks can have high viscosity and require high extrusion speeds to preserve cell viability. Screw-assisted processing is effective to assure the uniform distribution of cells or other bioactive components within the ink, while also providing higher force to drive the ink through the nozzle. Therefore, screw driven printheads can provide a higher force and faster extrusion speeds, while accommodating different materials with a range of rheological properties. A printhead driven with a screw system can actively mix the feed materials with a temperature control element surrounding the nozzle, and has a higher material deposition rate compared to FDM based printing [34]. Various screw-based printhead designs have been developed to meet specific application requirements (Fig. 2). For example, Kumar et al. integrated a screw-driven printhead into a computer numerical control (CNC) system, using a drill bit as the extrusion screw to facilitate precise flexible material deposition (Fig. 2A) [35]. The CNC system can potentially offer higher spatial resolution compared to the conventional stage controller. Zhou et al. introduced a multi-port screw printhead with four feeding inlets, enabling multi-material printing without the need for prior mixing or preprocessing (Fig. 2B). [36]. This approach is particularly advantageous for thermally sensitive materials. Leng et al. designed a conical screw geometry to improve extrusion of highly elastic and flexible materials, which are typically challenging to process using conventional 3D printing systems (Fig. 2C) [37]. There are still no bioprinting applications using these novel designs and only a few bioprinting studies have employed screw-driven printheads [38,40]. The main reason is that generally an extended mixing time is required to ensure effective mixing. Apart from the increased processing time, the additional stress exerted by the rotating screw complicates the printing of living cells [38]. Previous studies also indicate that screw-driven printing causes more damage to cells than pressure-driven printing (Fig. 2D) [38], motivating the development of advanced designs that mitigate this issue while retaining the benefits of effective mixing and high-viscosity extrusion. In this regard, Bhattacharyya et al. have designed a 3D printhead with a twin screw extruder of variable screw pitches and two feeding inlets (Fig. 2E), with which cells are fed in the bottom inlet to reduce the excessive mechanical stress applied from mixing through the long mixing distance [39]. The core part in this two-screw extruder printhead is a widely used part in industry, featuring effective mixing and heating of the processed materials. The two screws used can be co-rotating, counter rotating and intermeshing to different degrees [41]. The concept can be adapted to fulfill different biofabrication requirements. The design shown in Fig. 2F ensures homogenous mixing of the bioink with multiple components and minimizes sedimentation effects. Justino Netto et al. have introduced a modular printhead with co-rotating twin screw extrusion with band heaters wrapped around the active mixing part, with which materials in pellet or powder form could be mixed extruded for 3D deposition [20]. Furthermore, triple screw, quad screw and other multiple screw extruders are emerging as an improved venue for effective manufacturing polymer blends and composites compared to the conventional single or dual screw extruders [42]. Some other designs can be found in a recently published comprehensive overview of screw-based printhead technologies towards conventional material printing [34]. The relevant concept, and even the entire extruder assembly, could also be directly used as a printhead with a 3D positioning system for bioprinting. The higher mixing efficiency should enable the effective blending of biocomponents and materials within a short distance, reducing the stress applied to sensitive components. The feeding pot also allows convenient tuning of the bioink composition, even during printing, opening up the possibility to deposit gradients of biomaterials and cells.

The rheological properties of inks used with functional screw-driven printheads should follow the general design principles described in the previous section. Because such systems can exert higher forces on processed materials, bioinks with higher viscosities can be printed. However, when incorporating living cells, higher viscosity also results in increased shear stress, which may compromise cell viability or functionality, particularly for shear-sensitive cells such as stem cells and neurons. Therefore, bioinks used with screw-driven printheads should maintain a moderate viscosity to preserve cell activity, while leveraging temperature control systems and enhanced internal material convection to reduce effective viscosity during cell processing.

### 3.2 Core-shell printhead

**Fig. 3:**
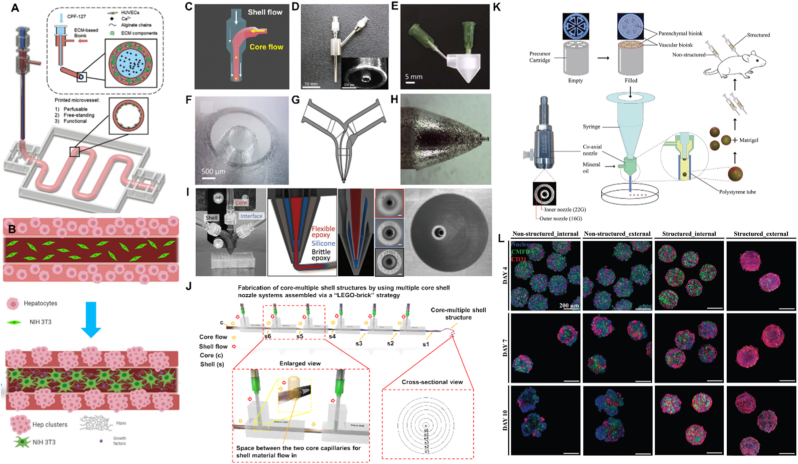
**Core-shell printing enabled tissue engineering and core-shell nozzle/needle designs for 3D fabrication.** (A) Schematic representation of an established vascular model through core shell printing [43]. (Adapted with permission from Ref. [43]. Copyright Wiley-VCH 2018). (B) Schematic representation showing a strategy for fabricating improved liver microenvironment through core shell fabrication [44]. (Adapted with permission from Ref. [44]. An open-access article under the terms of the Creative Commons CC BY license). (C) Schematic representation of a typical core-shell nozzle structure. (D) Core-shell nozzle fabricated by machining [45] (Adapted with permission from Ref. [45]. Copyright Wiley-VCH 2011). (E) Core-shell nozzle prepared by SLA based 3D printing and (F) its magnified tip structure [46]. (Adapted with permission from Ref. [46]. Copyright IOP Publishing Ltd 2019). (G) Design and (H) tip structure of a core-shell nozzle fabricated via 3D metal printing [47]. (Adapted with permission from Ref. [47]. Copyright IOP Publishing Ltd 2014). (I) Core-two-shell nozzle with corresponding cross-sectional and end views [48]. (Adapted with permission from Ref. [48]. Copyright Wiley-VCH 2018). (J) Stackable core-shell nozzle design enabling the flexible fabrication of structures with multiple shells [49]. (Adapted with permission from Ref. [49]. An open-access article under the terms of the Creative Commons CC-BY-NC-ND license). (K) A printhead developed by mounting a precursor cartridge on top of a core-shell nozzle, enabling the fabrication of multi-cell-laden spheroids. (L) The fabricated hepatic lobule-like structures exhibited improved structural integrity compared with unpatterned constructs [50]. (Figures K-L have been adapted with permission from Ref. [50]. An open-access article under the terms of the Creative Commons CC-BY-NC-ND license).

The core-shell (or co-axial) printhead has emerged as one of the most versatile and widely adopted functional printing technologies in recent decades, finding applications across diverse domains. This configuration enables the fabrication of printed structures with distinct internal and external compositions, and offers enhanced flexibility and programmability. In biomedical engineering, for instance, core-shell printheads facilitate vasculature engineering through the deposition of sacrificial core materials (Fig. 3A) [43] and enable complex tissue fabrication by incorporating distinct cell populations in the core and shell compartments (Fig. 3B) [44]. At the heart of this technology is the core-shell nozzle/needle, which integrates a coaxially aligned core and shell flow system (Fig. 3C). While some commercially available core-shell print nozzles exist [44], customized configurations can be fabricated via precision machining with coaxial alignment (Fig. 3D) [45], stereolithography (SLA) 3D printing (Fig. 3E and F) [46] or metal 3D printing (Fig. 3G and H) [47]. As a complement to the core-shell nozzle design for fabricating hollow channels, Feng et al. developed a nozzle with embedded needles that simultaneously generates holes during material extrusion, eliminating the need to extrude fugitive inks simultaneously [51]. However, this strategy requires inks with a high storage modulus to maintain the channel structures after deposition. To further expand functionality and versatility, multi-shell core nozzles have been introduced. Mueller et al. developed an advanced variation incorporating an additional interfacial layer between conventional core-shell components, increasing structural complexity and material compatibility (Fig. 3I) [48]. Moreover, Li et al. proposed a stackable nozzle design, allowing the fabrication of fibrous structures with multiple functional shell layers surrounding a central core (Fig. 3J) [49]. The core-shell setup is also a powerful tool for microbead fabrication, enabling the formation of spheroids once cells are encapsulated [49,52]. Especially, Hong et al. developed a printhead with a precursor cartridge mounted on a core-shell nozzle, enabling fabrication of patterned, multi-cell-type spheroids (Fig. 3K) [50]. The fabricated Hepatic-Lobule-Like structures exhibited improved structural integrity compared with unpatterned constructs (Fig. 3L). These advances underscore the transformative potential of core-shell printheads in expanding the capabilities of 3D bioprinting, particularly in the fabrication of hierarchical, multi-material structures, which is essential when building analogues of *in vivo* structures.

The rheological properties of inks used with core-shell printheads should follow the general design principles described in the previous section. However, when the shell layer is mechanically stable, the rheological constraints on the core material are substantially relaxed. Even low-viscosity, aqueous materials can be retained within the core when the shell is efficiently stabilized after extrusion [49]. As a result, cells encapsulated in the core can be effectively protected from excessive shear stress on the nozzle wall. Nevertheless, because the core diameter is typically small, processing highly viscous core materials may require elevated pressures, which could subsequently induce cell damage. In summary, shell materials should adhere to the general rheological requirements for 3D-printable inks, whereas greater flexibility is permissible in the selection of core materials.

### 3.3 Printhead with a magnetic field

**Fig. 4:**
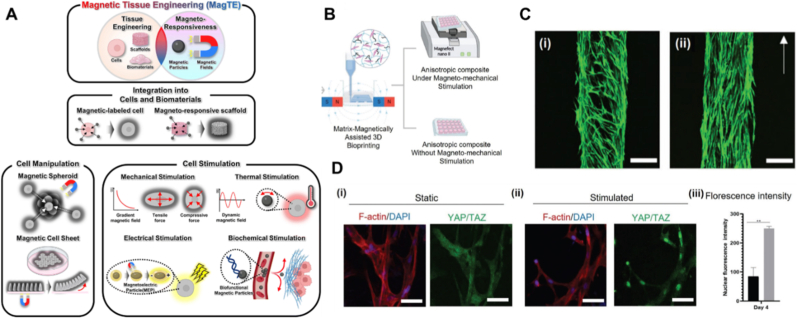
**Magnetic tissue engineering and a typical design of 3D printhead with an integrated magnetic field.** (A) An overview of magnetic tissue engineering, including cell manipulation and stimulation through integration of cells and magnetic materials [59]. (Adapted with permission from Ref. [59]. An open-access article under the terms of the Creative Commons CC-BY-NC license). (B) Magnetically assisted 3D bioprinting with bioink containing magnetic microfillers. (C) Cell culture in 3D bioprinted hydrogels with i) randomly-oriented magnetic microfillers and ii) aligned magnetic microfillers at day 21 (Scale bars 250 μm). (D) Magneto-mechanical stimulation promotes tendon phenotype differentiation of the embedded stem cells (Scale bars 50 μm) [60]. (Figures B-D have been adapted with permission from Ref. [60]. Copyright Wiley-VCH 2022).

Biocompatible magnetic materials have been utilized in tissue engineering to enable the multi-layer 3D assembly of cell laden microgels [53], production of organoids [54], coding of 3D living architectures [55] and to promote dense, oriented tissue formation [56]. Magnetic materials have also been incorporated into bioinks to improve control over 3D printed cellular structures [57,58]. In addition, embedded magnetic materials enable non-invasive stimulation through the forces they generate when exposed to external magnetic field (Fig. 4A) [59] and generate localized heat for hyperthermia therapy [61]. However, conventional extrusion printheads lack mechanisms for controlling embedded magnetic materials, which significantly restricts any post-printing manipulation.

Magnetic forces can be used to control the alignment of magnetic particles or anisotropic structures during 3D printing. For instance, by leveraging the directional influence of a magnetic field, it becomes feasible to align embedded ferromagnetic particles or responsive elements within the ink or matrix during extrusion, encoding spatially controlled magnetic domains directly into the printed structure. This approach is useful for the fabrication of anisotropic tissue constructs. Magnetic microfillers have been incorporated into bioinks and aligned during printing under an external magnetic field to provide guidance for cell growth (Fig. 4B) [60]. The 3D printed structure shows well aligned cells after 21 days of culture and can also be subjected to magneto-mechanical stimulation (Fig. 4B and C). This stimulation activates mechano-signaling pathways, thereby triggering tendon phenotype differentiation of the embedded stem cells (Fig. 4D).

More design parameters (actuation field, the strength of magnetization and domain patterns) can be introduced with the magnetic field assisted printhead to broaden the application spectrum of anisotropic tissue printing. The application of these mechanisms to bioprinting could open up the possibility to obtain magnetically transformable 3D bio-scaffolds. Magnetically active bioinks could be formulated using magnetic additives or magnetically responsive cells. In particular, when combined with newly developed magnetic nanotweezers [62], which would allow the mechanical stimulation within the 3D printed tissues.

The rheological properties of inks used with the printhead incorporating a magnetic field should follow the general design principles described in the previous section. The incorporation of magnetic particles generally increases the viscosity, yield stress and elastic modulus of bioinks through interactions with surrounding polymer networks [63]. However, excessively high particle loadings can induce elevated shear stress during extrusion and reduce printing resolution [64]. Iron oxide nanoparticles are the most widely used magnetic additives and are generally considered cytocompatible [64]. Common surface-engineering strategies, such as additional coatings, can further protect cells within the bioink and reduce the potential toxicity of magnetic particles. Printed constructs typically exhibit high cell viability immediately after printing; however, long-term biocompatibility largely depends on the retention, degradation and clearance of the particles. Widely used iron oxide nanoparticles can be degraded and incorporated into normal iron metabolism, with their superparamagnetic properties diminishing over time and their biodistribution shifting from the liver to the spleen *in vivo* [65]. In contrast, non-degradable magnetic particles or those prone to persistent aggregation may elicit chronic inflammatory responses. Magnetic particles used for magnetic bioink preparation should be carefully selected according to the intended application.

### 3.4 Printhead with an electric field

**Fig. 5:**
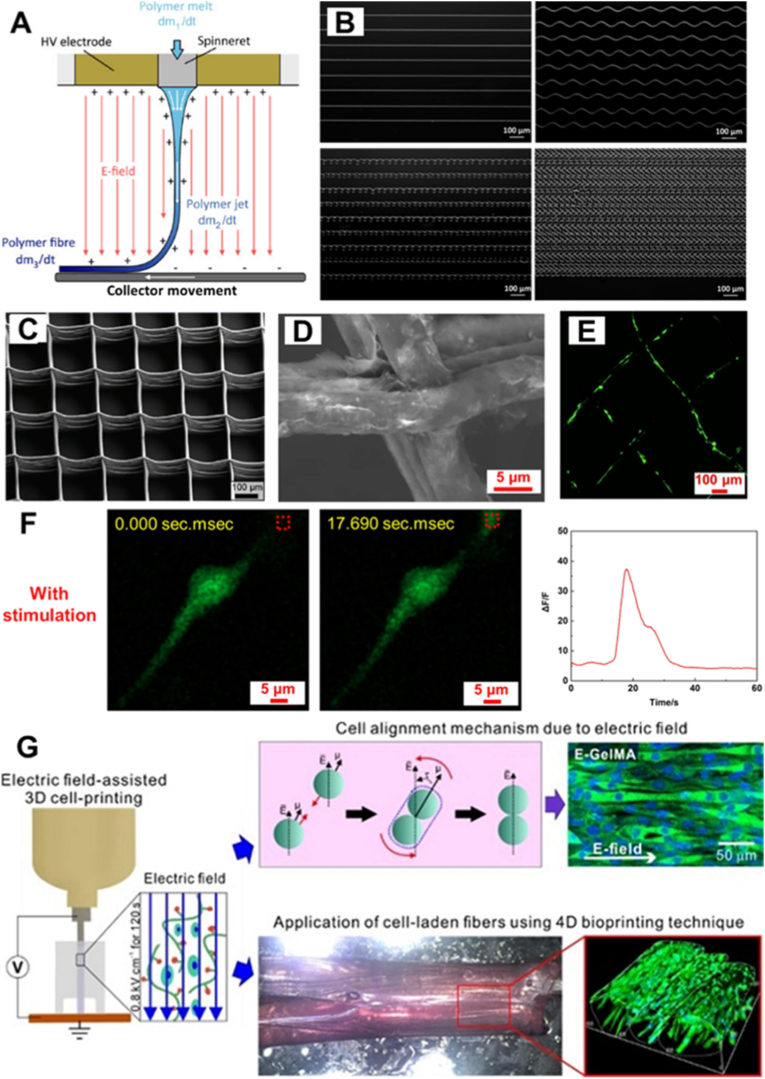
**Typical designs of printhead integrating electric fields.** (A) Schematic representation of the printhead incorporating an electric field [66]. (B) Different patterns printed by controlling the collector speed [66]. (C) Multilayer 3D scaffold printed with MEW [66]. (Fig. 5A–C were adapted with permission from Ref. [66]. An open-access article under the terms of the Creative Commons BY-NC-ND 3.0 license). (D) MXene coated MEW printed structure [67]. (E) Fluorescence image of SH-SY5Y cells growing along the MEW printed structure (Calcein: green) [67]. (F) Fluorescence imaging of a representative SH-SY5Y cell at various time points under red light stimulation, accompanied by a graph depicting the relative fluorescence intensity change within the region of interest (red dashed square) [67]. (Fig. 5D–F were adapted with permission from Ref. [67]. An open-access article under the terms of the Creative Commons CC BY license). (G) Schematic representation of electric field-assisted 3D cell printing, with inlets showing the mechanism and the resulting cellular structures [68]. (Adapted with permission from Ref. [68]. An open-access article under the terms of the Creative Commons Attribution License).

Similarly, the printed constructs can be subjected to an electric field by applying high voltage to the printhead while grounding the collector substrate or vice versa, giving rise to a technique known as melt electrowriting (MEW). In contrast to conventional electrospinning, which results in randomly deposited fibers, MEW utilizes a molten, electrically charged thermoplastic polymer that is precisely extruded and deposited under the influence of the electric field (Fig. 5A) [66]. Unlike materials printed under an external magnetic field, materials that are 3D-printable using an external electric field only need to be weakly electrically conductive or polarizable to enable jet formation and do not require encapsulation of additional functional particles. The applied electric field stabilizes and accelerates the polymer jet, enabling high fidelity control over fiber placement. By modulating parameters such as collector velocity (Fig. 5B), applied pressure, voltage and the nozzle-to-collector distance, complex three-dimensional architectures with microscale resolution can be fabricated (Fig. 5C) [66]. The high precision and reproducibility of MEW allow the creation of scaffolds with structural features at the cellular or even subcellular scale, which is essential for recapitulating native extracellular matrix and facilitating the spatial guidance of cell behavior. For instance, Li et al. enhanced the bioactivity of MEW-fabricated polycaprolactone (PCL) scaffolds by coating them with two-dimensional MXene nanosheets, imparting not only improved cellular affinity and 3D spatial control of neuronal cells, but also a photothermal capability for the remote modulation of neuronal activity under light stimulation [67] (Fig. 5D). Additionally, Yang et al. explored electric field-assisted 3D printing of cell-laden, hydrogel-based bioinks [68]. The printed cells exhibited improved maturation; however, no well-defined 3D scaffold was demonstrated (Fig. 5D). It remains challenging to control polymer jet stability under an electric field and further efforts are needed to achieve stable printing conditions. Further details on electric field assisted 3D printing can be found in a recently published review paper [69].

The materials used with printheads incorporating an electric field are generally thermoplastics. Their printability relies on thermal processing and the drawing effect induced by the electric field prior to deposition. These materials must exhibit suitable viscosity to pass through the nozzle after melting within the cartridge and viscosity tuning through the addition of solvents to assist extrusion is also possible [70]. In contrast, living cells can only be printed using hydrogel-based inks, whose rheological properties should follow the general design principles described in the previous section. Further work is required to explore direct cell printing strategies in this direction.

### 3.5 Printhead with an acoustic field

**Fig. 6:**
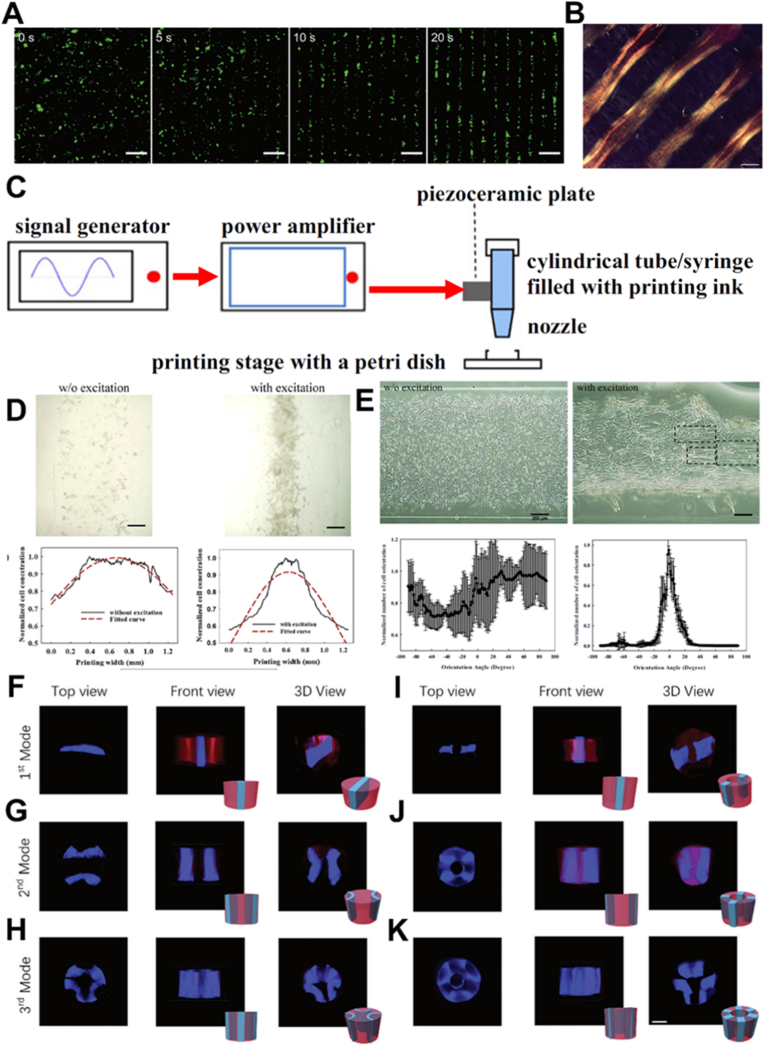
**Tissue engineering assisted by acoustic field and a typical design of printhead with an integrated acoustic field.** (A) Fluorescent microscopy of chondrocytes (green) patterned by a 6.7 MHz ultrasound standing wave at different time points (Scale bars = 200 μm) [71]. (B) A patterned cartilage tissue was formed after 35 days culture (50 μm) [71]. (Fig. 6A and B were adapted with permission from Ref. [71]. An open-access article under the terms of the Creative Commons CC BY License). (C) Schematic representation of the printhead incorporating an acoustic field, achieved through the integration of piezoceramic plate [73]. Functional validation of the acoustically assisted printhead: (D) cell accumulation within printed structures; (E) cell aligning with the excitation of acoustic field [73]. (Fig. 6C–E were adapted with permission from Ref. [73]. An open-access article under the terms of the Creative Commons Attribution 4.0 International License). (F-K) Different cellular patterns formed within fibers and tubules under different acoustic modes (Scale bars = 500 μm) [74]. (Fig. 6F–K were adapted with permission from Ref. [74]. An open-access article under the terms of the Creative Commons CC BY License).

Likewise, printed materials with sufficient acoustic contrast can be actively manipulated using external acoustic fields, which offer a non-contact, biocompatible, additive free and highly controllable method for spatially organizing particles and cells within printed constructs. Acoustic waves can induce the migration and accumulation of suspended components at pressure nodes in real-time, enabling precise positioning without direct mechanical interference. This phenomenon minimizes the risk of contamination as well as perturbation to delicate biological components. Acoustic fields are particularly advantageous for engineering cellular architectures and conductive composites, where relative positioning between individual components is critical for facilitating cell-cell interactions [71] or establishing continuous conductive pathways [72]. Especially for acoustic field assisted cell patterning, mature tissue was formed after long-term culture even though the cells were not fully connected in the initial cellular patterns (Fig. 6A and B) [71]. A representative implementation of acoustic fields in 3D bioprinting was presented by Sriphutkiat et al., who introduced an acoustically augmented printhead by integrating a piezoceramic plate onto a glass dispensing nozzle to generate acoustic waves at specific resonant frequencies during extrusion process of cell laden bioinks (Fig. 6C) [73]. When activated during printing, the acoustic field directed cells to aggregate centrally within the deposited filaments, resulting in centrally aggregated cellular distributions irrespective of cell type (Fig. 6D) [73]. In contrast to the more loosely and heterogeneously distributed cells observed in constructs printed without acoustic stimulation, the acoustically patterned cells exhibited pronounced cellular alignment along the printing direction (Fig. 6E) [73], highlighting the potential of utilizing acoustic fields not only for aggregation but also for directional organization. However, in addition to the parallel cellular alignment observed in biology, radial and other complex patterns are also found in vessels and liver tissues, playing important roles in their respective functionalities [75,76]. To further expand the potential of printing with an acoustic field, Yin et al. investigated the patterns generated using different capillary shapes, materials and applied acoustic fields [74]. Various patterned cell-laden fibers and tubules were continuously produced with this setup using different acoustic molds (Fig. 6F–K), but no 3D scaffold has been fabricated with this system to date. Such methodologies are especially promising for applications in neural tissue engineering, cardiac patch development and composite bioelectronics, where microscale precision and internal organization are crucial.

Bioinks used with printheads incorporating an acoustic field should meet the general rheological requirements for 3D printing; however, their viscosity should not be excessively high, as this would inhibit cell movement under acoustic force exposure. Successful patterning also requires efficient acoustic coupling of ultrasound, and therefore the bioink should have an acoustic impedance similar to that of water.

### 3.6 Printhead with an adaptive outlet

**Fig. 7:**
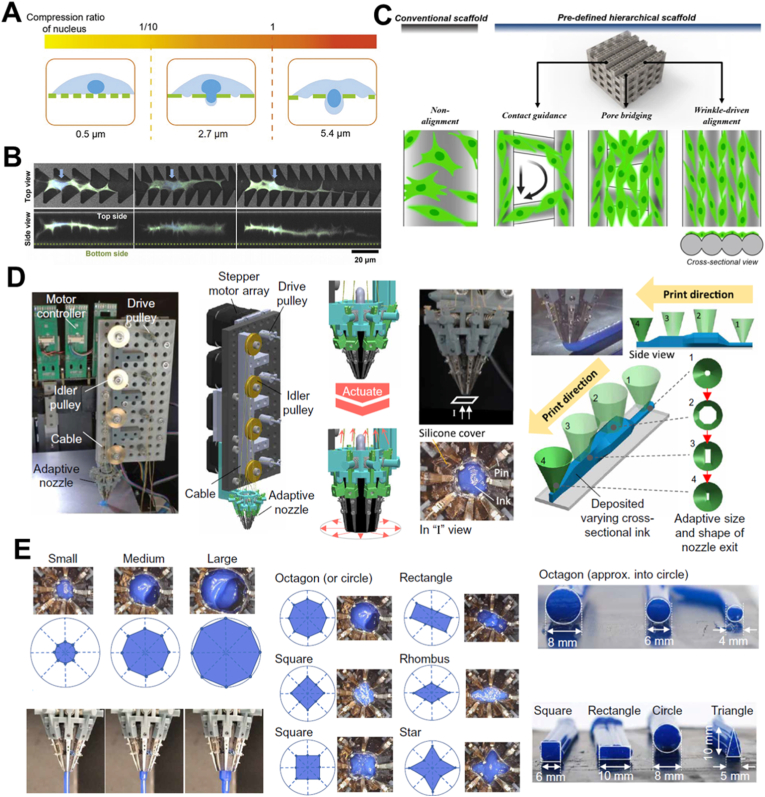
**Geometries within the scaffold influence cell behavior and a typical printhead design equipped with an adaptive nozzle.** (A) Pore sizes in the scaffold influence cell infiltration of cells [79]. (Adapted with permission from Ref. [79]. Copyright Wiley-VCH 2024). (B) Cell morphology and migration can be influenced by the scaffold geometries [80]. (Adapted with permission from Ref. [80]. An open-access article under the terms of the Creative Commons Attribution 4.0 International License). (C) Cell morphology and alignment are influenced by scaffold local designs [81]. (Adapted with permission from Ref. [81]. An open-access article under the terms of the Creative Commons Attribution-NonCommercial-NoDerivatives 4.0 International License). (D) Overview of the 3D printer equipped with the adaptive nozzle with schematic representation and photos of the printhead design. (E) Photo and schematic representation showing the nozzle orifice shape/size changing during printing, and also photos showing filaments with different shapes obtained with different orifice size of the nozzle [82]. (Adapted with permission from Ref. [82]. An open-access article under the terms of the Creative Commons Attribution 4.0 International License).

Conventional 3D printheads are generally equipped with a nozzle of fixed outlet shape, which limits the printing flexibility during printing. Generally, a smaller nozzle orifice results in higher resolution but slower printing speeds, while a larger nozzle orifice conversely achieves higher printing speeds with reduced printing detail. Furthermore, studies on 3D printing cement or mortar have indicated that the nozzle shape in the printheads have significant influence on the printed structure integrity and morphology [77,78], which also applies to cellular scaffold fabrication. Different pore shapes and sizes, as well as variations in the solid regions of a scaffold, influence cell infiltration, migration, alignment and development (Fig. 7A–C) [[79], [80], [81],^83^], which suggests that these technologies can be of interest in 3D bioprinting. To address this challenge, Kang et al. have introduced an adaptive nozzle capable of changing outlet shapes and sizes, made of flexible, pressure-resistant membrane controlled through eight independently, tendon driven pins (Fig. 7D) [82]. Continuous gradients could be achieved through the adaptive design to enhance the printed structure density and contour precision (Fig. 7D) [82]. Not only the extruded strand size, but also the strand geometries can be well controlled through the design (Fig. 7E), significantly enlarging the potential for relevant applications [82]. This concept could find wide application in large tissue/organ bioprinting, where an optimized printing strategy could be implemented to achieve a high resolution within a reasonable total printing time.

The rheological properties of inks used with printheads featuring an adaptive outlet should follow the general design principles described in the previous section. However, when the outlet diameter becomes too small, ink viscosity should be reduced to avoid the excessive pressures required to drive materials through the nozzle, particularly when working with cell-laden inks.

### 3.7 Printhead with active and passive mixing nozzles

**Fig. 8:**
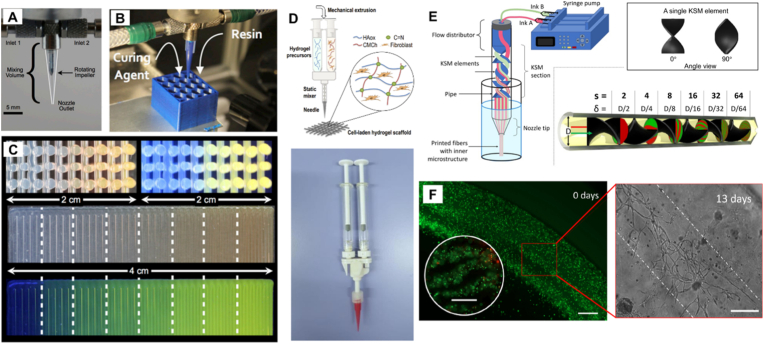
**A typical design of printhead with an impeller-based active mixing nozzle with examples of static mixing printhead for bioprinting.** (A) The design of the nozzle. (B) 3D printing two chemically active components. (C) 3D printed compositional gradient structures by simply varying the flow rate rations of the two inlet channels [85]. (Fig. 8A–C were adapted with permission from Ref. [85]. An open-access article under the terms of PNAS open access option). (D) A static mixing printhead used for reactive bioink printing [86]. (Adapted with permission from Ref. [86]. An open-access article under the terms of Creative Commons Attribution (CC BY) license). (E) A printhead designed for continuous chaotic printing incorporating multiple Kenics static mixer elements. (F) Chaotically bioprinted structures exhibit high cell viability after printing (left image with an inlet, scale bars: 500 μm) and the original structures are preserved during culture (right image, scale bar: 200 μm). (Figures E-F have been adapted with permission from Ref. [87]. An open-access article under the terms of Creative Commons Attribution 4.0 license).

Dual or multimaterial 3D printing is increasingly in demand across diverse disciplines, as the integration of multiple components is critical for constructing complex architectures with prescribed compositional gradients. Especially in tissue engineering, a continuous gradient of porosity, biochemical composition and mechanical properties in the scaffold material could better mimic natural counterparts, providing an advantageous platform for tissue regeneration [84]. However, effectively mixing materials in low Reynolds number fluid flows at small volumes and short residence times poses a significant challenge due to restricted diffusive transport. To address this challenge, Ober et al. have designed a printhead with an active mixing nozzle that can mix materials efficiently at a low ratio of channel length l over hydraulic diameter d (l/d) and high Péclet number (*Pe*) with sufficiently high impeller speeds [85]. Compared to screw-driven printhead, this design offers greater controllability over the input materials while providing efficient mixing capability. The impeller-based active mixer is composed of two inlets for introducing two different materials and an impeller made from a reamer to fit in a normal conical nozzle (Fig. 8A). The mixer could print dual materials chemically active with unstable rheological properties by overcoming the kinetic constrains through the fast mixing of the components in the nozzle right before deposition (Fig. 8B). By continuously varying the inlet flow rates, structures with uniform compositional gradients can be printed (Fig. 8C). Similarly, Kokkinis et al. introduced a two-component dispensing system with a static disposable mixer, enabling compositional ratios across a broad range (17-83 vol%) and demonstrating the capacity to fabricate structures with spatially programmed mechanical gradients [88]. A similar static mixing tool was also employed in the 3D bioprinting of reactive hydrogel-based bioinks (Fig. 8D), enabling the printing process to be well matched with the ink's crosslinking kinetics. [86]. The mixing components are not only used to generate material gradients or to obtain uniform bioink outputs, they can also be employed to produce extensive lamellar microstructures within printed constructs. The concept of continuous chaotic printing was demonstrated by designing a printhead incorporating multiple Kenics static mixer elements [89], enabling the fabrication of fibers with distinct internal microstructures (Fig. 8E) [87]. The resulting striation patterns were maintained during the tissue culture process (Fig. 8F), highlighting the potential of this approach for creating cell-laden fibers with multilayered microstructures. These advances highlight how active and passive microscale mixing strategies can overcome long-standing kinetic and rheological barriers in dual or multimaterial printing, thereby enabling effective in situ bioink formulation, versatile 3D biofabrication of gradient scaffolds, and the creation of multi-material and multi-lamellar structures.

Because the rheological properties of inks can change during processing within printheads that incorporate mixing capabilities, there are fewer rheological constraints on the individual components prior to mixing. However, after mixing, the resulting ink should exhibit shear-thinning behavior and a short recovery time to restore its solid-like state after deposition. Viscosity also plays a critical role in mixing efficiency. For different mixing mechanisms, the viscosity compatibility of the individual components should be carefully studied to ensure effective mixing before deposition.

### 3.8 Multi-nozzle printhead

**Fig. 9:**
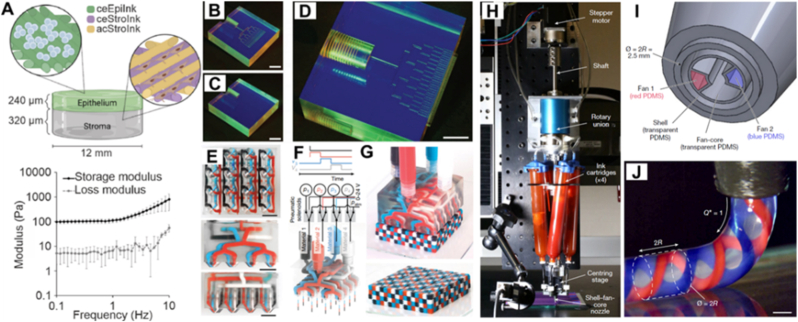
**Multi-nozzle printheads are required for improved tissue engineering with examples of multi-nozzle printheads.** (A) Multiple cell types and biomaterials are required in 3D bioprinting to better replicate native tissues. [90]. (Adapted with permission from Ref. [90]. An open-access article under the terms of Attribution 4.0 International Deed). (B) CNC-milled acrylic block with microvascular channel networks. (C) Unpatterned acrylic block used for sealing. (D) Welded nozzle assembled from components shown in (B) and (C) [91]. (Fig. 9B–D were adapted with permission from Ref. [91]. Copyright Wiley-VCH 2013). (E) Top and side views of a 4 × 4 nozzle, four-material 2D printhead (scale bars, 10 mm). (F) Schematic representation illustrating the operational mechanism of the nozzle. (G) The 4 × 4 nozzle, four-material 2D printhead used for voxelated matter 3D printing [92]. (Fig. 9E–G were adapted with permission from Ref. [92]. Copyright Springer Nature 2019). (H) Setup of the rotational MM3D system. (I) Design of a multi-material shell-fan-core nozzle tip. (J) PDMS-based strand deposited from the nozzle shown in (H) [93].(Fig. 9H–J were adapted with permission from Ref. [93]. Copyright Springer Nature 2023).

The active mixing printhead described in the preceding section enables the co-processing of two materials. However, to fully exploit the potential of multi-material additive manufacturing, a printhead capable of handling a larger number of materials is required. Native tissues are highly heterogeneous, with diverse cell types and extracellular matrix components. For example, corneal 3D bioprinting requires multiple cell types and inks to more accurately replicate the structure of native corneal tissue (Fig. 9A) [90]. Most current multi-material 3D bioprinting approaches rely on sequential use of multiple printheads during printing, which is time consuming and can introduce misalignment in the printed scaffold. To overcome these limitations, multi-nozzle printheads have been developed. Hansen et al. addressed this challenge by fabricating multi-nozzle arrays using CNC milling to precisely pattern poly(methyl methacrylate) (PMMA) blocks (Fig. 9A–C) [91]. This scalable design enables parallel deposition of multiple materials with channel diameters as small as 10 μm. Notably, the multi-nozzle configuration can significantly enhance printing throughput, as for instance with a 64-nozzle array, while simultaneously reducing alignment errors. However, the bifurcation-based architecture necessitates high driving pressures, particularly in multilayered designs; for instance, a 64-nozzle microvascular network employing a shear-thinning ink demands pressures approaching 430 MPa.

To further expand the resolution and flexibility of multi-material deposition, Skylar-Scott et al. introduced a voxelated multi-material multi-nozzle 3D (MM3D) printing system, which enables rapid in-nozzle material switching via a bank of high-speed pneumatic solenoids (Fig. 9D–F) [92]. This voxelated strategy allows the material composition to be programmed, as well as function and spatial organization. The system's versatility was demonstrated by fabricating complex structures such as foldable origami architectures and soft robotic actuators. Pushing the boundaries toward subvoxel precision, Larson et al. developed a rotational MM3D printing approach capable of producing helically structured filaments with tunable helix angles, layer thicknesses and interfacial geometries (Fig. 9G–I) [93]. This rotational design facilitates the fabrication of helical dielectric elastomer actuators featuring independently addressable conductive channels, as well as hierarchical lattice structures composed of mechanically distinct spring and matrix domains. Both designs shed light on 3D printing of complex architectures which should also lend themselves to bio-printing with well-programmed cellular architectures and distributions of bioactive factors, which are needed to recreate conditions found *in vivo*.

However, several issues still need to be addressed for multi-nozzle printheads. First, high-resolution multi-nozzle printheads are prone to clogging due to their small nozzle diameters, particularly when processing high-viscosity inks or cell-laden bioinks. Strategies such as submerging the nozzle tip in solvent to prevent ink drying or integrating advanced purging cycles during printing can effectively mitigate nozzle clogging. Second, when printing multiple materials, there is a risk of cross-contamination due to leakage or diffusion between adjacent channels. To minimize this risk, the printhead should be tested before using and the inks used in different channels should be carefully evaluated and strategically selected. For highly diffusible inks, protective coatings introduced through core-shell printing or subsequent post-printing treatments are necessary. Finally, as standardization of 3D printers is essential for broader applications, the development of robust calibration protocols is particularly important to ensure precise control of flow rates across different channels and to synchronize multiple material flows when required.

The rheological properties of inks used with multi-nozzle printheads should follow the general design principles described in the previous section. Because some nozzles have very small internal diameters, ink viscosity should be sufficiently low to avoid the need for excessive extrusion pressure. For voxelated printing strategies, the ink should exhibit a higher storage modulus to support the surrounding structures after deposition.

### 3.9 Multi-material multi-nozzle adaptive printhead

**Fig. 10:**
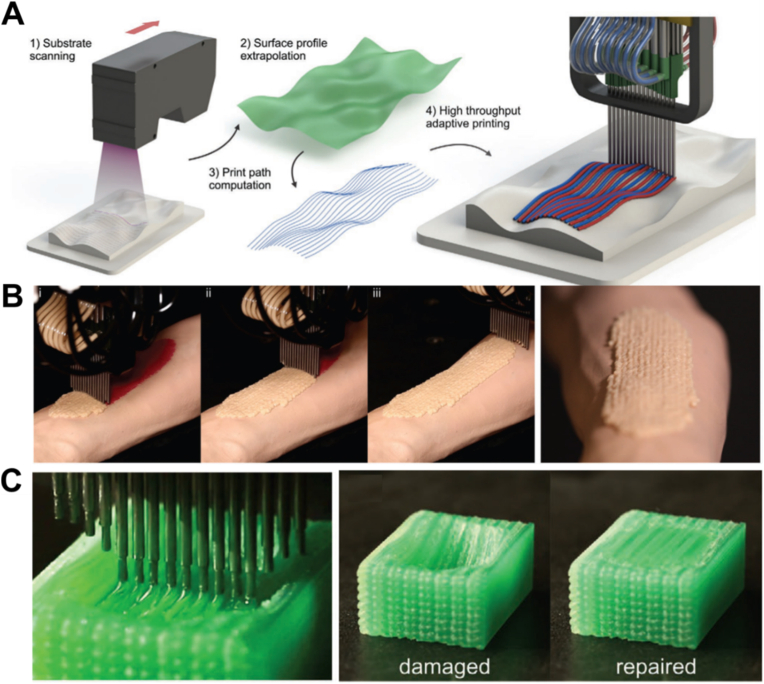
**A typical design of adaptive printhead with multi-nozzles for multi-material and printing on uneven surfaces.** (A) Schematic representation of the 3D printing workflow for using the multi-material multi-nozzle adaptive printhead. (B) Application of the printhead towards wound healing. (C) Application of the printhead towards damage repair [94]. (Fig. 10A–C were adapted with permission from Ref. [94]. Copyright Wiley-VCH 2022).

Multiple materials could be 3D printed with the multi-nozzle system introduced in the previous section, but the printing substrate is restricted to planar morphologies. To fully unleash the potential of these printheads, especially for biomedical applications, printing on irregular, conformal substrates is required. Uzel et al. have introduced a multi-nozzle printhead with each nozzle can be positioned in an interdigitated manner [94]. The morphology of substrate is first measured and printing path of each nozzle is subsequently extrapolated from the measured surface profile and computed (Fig. 10A). The potential of this approach in biomedical applications has been demonstrated by the conformal printing of gelatin-based inks across wound regions (Fig. 10B) and by the precise deposition of polyacrylate-based inks with tailored mechanical properties to restore structural integrity in a defect model (Fig. 10C). An adaptive 3D bioprinting system with multiple printheads has been used for wound healing *in vivo* testing and the integrated imaging system allows precise printing of cells and materials on complex substrates, promoting fast wound healing with accelerated re-epithelialization and reduced contraction [95]. The adaptive printhead is an important milestone in smart 3D printing, particularly for enabling the future use of 3D printing in clinical applications during surgery.

Since the extrusion components are identical to those of multi-nozzle printheads, the discussion of ink design can refer to the corresponding part in the previous section.

### 3.10 Hybrid printhead

**Fig. 11:**
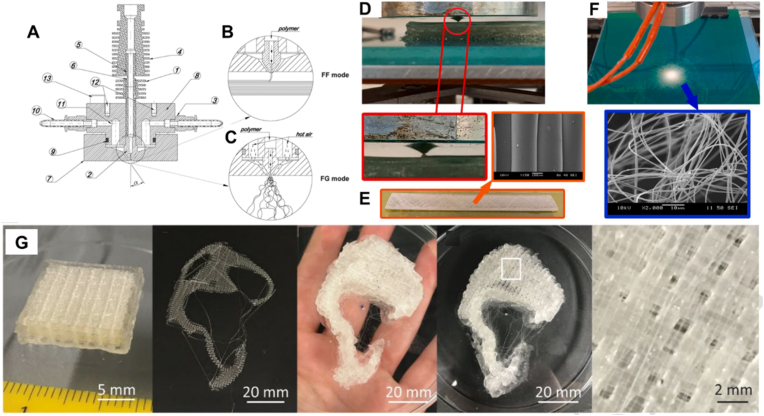
**An example of hybrid printhead with integrated nozzle: Extrusion and melt-blowing.** (A) Schematic representation of the nozzle design with (B, D, E) showing the operation modes of filament fusion and (C, F) showing fiber generation with corresponding SEM images of generated structures [97]. (Adapted with permission from Ref. [97]. An open-access article under the Creative Commons CC-BY-NC license). (G) Hybrid scaffold 3D printed with extrusion printing of hydrogel and MEW of PCL, showing potential for supporting auricular cartilage regeneration [98]. (Adapted with permission from Ref. [98]. Copyright Elsevier 2021).

The incorporation of nano- and micro-fibrous features into 3D-printed constructs offers significant opportunities to enhance the mechanical performance and tailor compositional properties, while providing instructive cues for cell growth within a mechanically stable matrix [96]. Conventionally, the fabrication of such hierarchical architectures requires multiple sequential processing steps, increasing cost and reducing precision, and often compromising sterility when printing scaffolds for cell culture. To overcome these limitations, Kara et al. developed an integrated printhead capable of fabricating constructs that combine nano-/micro-fibers with solid or infill layers in a single manufacturing step [97]. The core component of the printhead is an extrusion and melt-blowing nozzle (Fig. 11A–C), which can either fuse the filament for direct deposition (Fig. 11D and E) or melt-blow the extruded strand into ultrafine fibers using hot air (Fig. 11F). Fiber diameters can be precisely tuned by adjusting the nozzle orifice diameter and hot air velocity, achieving dimensions as small as 300 nm. By integrating extrusion-based and fiber-spinning techniques, this hybrid printhead enables the fabrication of hierarchical architectures with enhanced structural and functional properties, while exemplifying a promising strategy for next-generation multifunctional printhead design and application. Recent studies have demonstrated that hybrid scaffolds composed of chondrocyte laden hydrogels and microfibers show potential for supporting auricular cartilage regeneration [98]. However, when such scaffolds are fabricated using two separate printheads within a single printing station, switching between printheads can introduce misalignment. Employing a single hybrid printhead that eliminates the need for printhead switching can mitigate this issue and significantly accelerate the printing throughput. Further development of hybrid printheads that integrate multiple printing modalities through thoughtful design is therefore essential to enhance the efficiency and versatility of biofabrication.

The rheological properties of materials or inks used in hybrid printheads should comply with the requirements of all printing modalities. For the specific requirements of each modality, the discussion of ink design can refer to the previous sections.

### 3.11 Hand-held and robotic arm based printheads

**Fig. 12:**
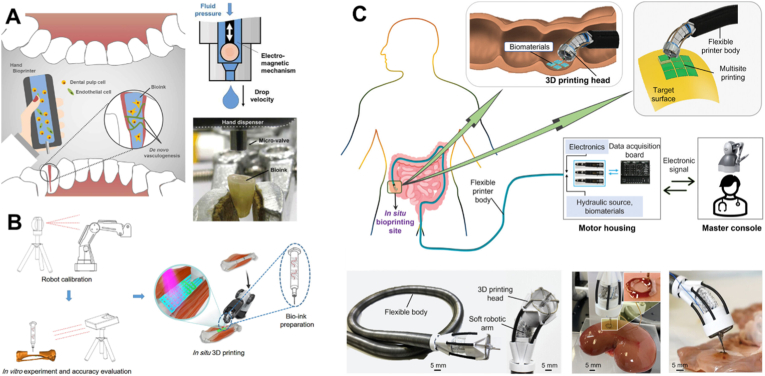
**Hand-held and robotic arm based printheads for in situ 3D bioprinting.** (A) Hand-held 3D bioprinter was used for vascularized dental pulp tissue bioprinting [99]. (Adapted with permission from Ref. [99]. Copyright Taylor & Francis 2019). (B) A printhead integrated with a robotic arm for in situ repairing large bone defects with bioinks [100]. (Adapted with permission from Ref. [100]. An open-access article under the Creative Commons CC-BY-NC-ND license). (C) A printhead integrated with a small flexible robotic arm is possible for endoscopic surgery through bioprinting in internal tissues or organs [101]. (Adapted with permission from Ref. [101]. An open-access article under the Creative Commons Attribution License).

Desktop-based 3D bioprinters are the most widely used systems, but they are difficult to use for *in vivo* 3D bioprinting, because of the limitations imposed by the fixed printing platform and the restricted movement space of the printing arms. Ideally, a printhead suitable for *in vivo* bioprinting would directly deliver cells and bioactive factors in situ in a geometrically matched 3D form, while avoiding the risks associated with the conventional print-culture-transplantation strategy. Hand-held 3D bioprinters allow surgeons to use a portable mini-printer in the operating room and deposit bioink in situ on demand (Fig. 12A). However, hand-held printing accuracy then largely depends on the user's skill and experience, which may introduce variability in treatment outcomes. To improve controllability, robotic arms have been introduced to guide the movement of printheads while maintaining flexible motion trajectories (Fig. 12B) [100]. In particular, robotic-arm trajectories can be precisely planned and guided using advanced integrated scanning and imaging systems [102]. However, both handheld printheads and large robotic-integrated printheads are limited to printing at or near the skin surface or in regions with large accessible operation areas, restricting their use in internal tissues or organs. To address this limitation, a printhead connected to a thin, flexible robotic arm with a high degree of freedom has been developed (Fig. 12C) [101]. This flexible-arm-based 3D printer can perform endoscopic surgery and deposit bioinks onto uneven internal substrates. However, this design is not yet fully automated and still requires human intervention for maneuvering. The material-delivery mechanisms used in these printheads include pneumatic systems [99,103] and pistol-type driving systems [101,104,105]. Since this is a newly emerging area, most printheads reported so far have relatively simple designs, such as straightforward single-nozzle extruders [[99], [100], [101],^104^], along with one dual-nozzle design [105] and one core–shell nozzle design [103]. However, these designs generally lack interactive guidance in complex, dynamic biological environments. Incorporating intraoperative data acquisition and analytics into printhead design would enable adaptive performance during surgery. Further advances, especially by integrating real-time imaging and mapping modalities, in printhead engineering are needed to accelerate progress toward surgical treatment strategies.

The inks used with these printheads are generally hydrogel-based and should follow the general design principles described in the previous sections.

## 4 Summary and outlook

Extrusion-based 3D printing has emerged as one of the most widely adopted additive manufacturing strategies over the past decades. Owing to its simplicity and versatility, it has been increasingly employed in biomedical engineering, soft robotic development and smart device fabrication. This printing approach imposes some rheological constraints on the materials, but can be used to print a broad spectrum of material candidates suitable for fabrication, which is especially useful for biofabrication.

The basic architecture of an extrusion-based 3D printer is relatively straightforward compared to other 3D printing platforms, typically consisting of a material driving system, a temperature control module and a dispensing unit. Successful printing relies heavily on the rheological properties (e.g., viscosity, elastic modulus, yield stress, etc.) and solidification mechanism of the ink, which influence recovery behavior and govern both processability and printing resolution. Passing through the narrow orifice of a nozzle or needle can impose substantial shear stresses, which may compromise the structural integrity and viability of embedded biological components when printing cell-laden bioinks [12]. Moreover, extrusion-based systems often provide limited control over internal material composition and generally require planar substrates for material deposition. When multiple materials need to be printed, this approach necessitates a change of the printhead, which may cause misalignment between the printed parts and prolong the overall time required for printing.

To address these limitations and extend the capabilities of extrusion printing, advanced functional printheads have been developed. These innovations enable the change of material composition during the printing process and often feature multiple inlet channels for co-extrusion of different inks, integrated mixing modules to generate homogeneous or spatially patterned constructs and programmable external stimuli (e.g., magnetic, electric or acoustic cues) to induce component alignment, phase transitions, assembly or crosslinking. Such advances significantly broaden the scope of extrusion-based 3D printing, which are needed for next-generation biofabrication. Different functional printhead designs and their applications are summarized in Table 1. In general, these printheads are derived from the basic extrusion-based 3D printing architecture, with additional features incorporated to enhance material processing before or during ink deposition. These added functionalities provide distinct advantages, such as in situ mixing to improve bioink preparation (e.g., functional screw-driven printheads and printheads with active or passive mixing nozzles), advanced manipulation of encapsulated materials (e.g., printheads incorporating magnetic, electric, or acoustic fields), novel engineering of extruded structures (e.g., core-shell printheads, multi-nozzle printheads, multi-material adaptive printheads and hybrid printheads), and improved adaptability for direct clinical applications (e.g., handheld printheads and robotic arm-based printheads). At the same time, these added features may introduce new design challenges. For example, the inclusion of screws or internal mixing structures can complicate cleaning and sterilization. However, this issue can be mitigated through modular designs with low-cost, replaceable components. A more detailed analysis of each printhead type is provided in Table 1.

**Table 1:** Summary of emerging novel printhead designs, their advantages, limitations over conventional extrusion-based 3D printer printheads, potential biomedical applications and key considerations for improvement.

Design	Advantages	Limitations	Bio-applications	Key considerations	Reference
Functional screw driven printhead	➢ Effective mixing of printed materials during extrusion ➢ Higher extrusion force ➢ Precise material flow control with a potentially higher print speed limit ➢ Possibility of real-time changes to the printing material composition ➢ Multiple screw designs for different purposes	➢ Additional shear stress applied by the screw blades during cell printing ➢ Difficult to clean and maintain	➢ In situ bioink composition control.	➢ Novel screw blade and inlet design to reduce the shell stress generated during cell printing ➢ Thermal gradient to assist printing and preserve cell activity ➢ Modular and low-cost, replaceable components for easy replacement of different parts	[20,[35], [36], [37], [38], [39]]
Core shell printhead	➢ Distinct compositions and properties of printed strands ➢ Possibility of fabricating hollow structures ➢ Cell encapsulated in the core part can be protected from shear stress ➢ Functional spheroids fabrication	➢ Easy to clog and damage ➢ Difficult to clean and maintain	➢ Co-printing of multiple cell types ➢ Vasculature engineering ➢ Controlled drug release ➢ Controlled microenvironment for cells	➢ More effective cleaning strategy development ➢ Cheap and more versatile alternatives development	[[43], [44], [45], [46], [47], [48], [49], [50], [51]]
Printhead with a magnetic field	➢ Improved control during and after printing ➢ 4D printing by controlling the printed magnetic domains in the structure	➢ Magnetic micro- or nanoparticles must be included in the ink, which may negatively influence cell activity ➢ Magnetic particles may aggregate and clog the nozzles	➢ Cell patterning ➢ Noninvasive cell stimulation.	➢ Development of inks containing magnetic materials with low acute and chronic toxicity ➢ Programmable magnetic field during printing to enable more advanced applications of printed living structures	[57,58,60]
Printhead with an electric field	➢ High printing speed ➢ Improved printing precision and resolution ➢ Property tuning of the printed material	➢ High voltage is required for the experiment ➢ Achieving stable printing can be challenging ➢ It is mainly used for thermoplastic materials, with very limited research on hydrogel-based materials	➢ Cell and material alignment ➢ Assisting formation of neuronal circuits	➢ Improved setups for printing cell-laden hydrogels ➢ Effects of high voltage on cell activity need investigation	[[66], [67], [68]]
Printhead with an acoustic field	➢ Contactless patterning of particles within the printing strand ➢ Enhanced ink mixing and homogenization ➢ No additives needed	➢ Only relatively large feature sizes can be achieved ➢ Limited material choices due to the requirement for low-viscosity inks and matched acoustic impedance ➢ Potential thermal effects	➢ Control of cell patterning within the printed construct	➢ Improved setups to enable a wider range of printable materials and finer feature resolution	[73,74]
Printhead with an adaptive outlet	➢ Improved controllability of printing resolution, speed and strand cross-sectional shape	➢ Complex design and difficult standardization	➢ Possible to optimize printing strategies for large tissues/organs	➢ Simplify the setup to promote standardization and broader applications	[82]
Printhead with active and passive mixing nozzles	➢ Enhanced mixing efficiency ➢ Complex architectures printing with prescribed compositional gradients	➢ Increased shear stress near the impeller or mixers ➢ Difficult cleaning and sterilization ➢ Complex design and poor standardization	➢ Fabrication of bio-scaffolds with gradients ➢ Printing of delicate bioinks	➢ Improve the setup by introducing modular mixing elements with optimized designs to reduce shear stress	[[85], [86], [87], [88]]
Multi-nozzle printhead	➢ High throughput 3D printing ➢ Improved spatio-temporal control of printed materials	➢ High risk of clogging and difficult to clean ➢ Complicated design and poor standardization ➢ Possible cross-contamination between channels due to leakage and diffusion	➢ Programmable complex tissue printing with multiple cells and biomaterials ➢ High throughput fabrication of 3D cellular structures	➢ Introduce advanced cleaning cycles to prevent clogging ➢ Incorporate shell flow or advanced coatings to prevent cross-contamination	[[91], [92], [93]]
Multi-material multi-nozzle adaptive printhead	➢ All the advantages of multi-nozzle printhead listed above with capability to print on substrates that are not flat	➢ All the disadvantages of multi-nozzle printhead listed above ➢ Pre-print path computation is needed	➢ All the applications of multi-nozzle printhead listed above with potential to be used in clinical and translational settings	➢ All the key considerations of multi-nozzle printhead listed above ➢ Incorporation of a real-time scanning and printing guidance system	[94]
Hybrid printhead: Extrusion and melt-blowing combined printhead	➢ Efficient 3D printing scaffolds with different strand diameters ➢ With a reasonable combination of different printheads, the hybrid printhead should demonstrate the advantages listed above for each individual printhead	➢ Complicated design and difficult for cleaning	➢ Hierarchical scaffold fabrication to support cells	➢ Modular design and easy configuration and cleaning	[97]
Hand-held printhead	➢ Portable and easy for overall device sterilization ➢ High degree of freedom for printing movement ➢ Possible to print on different surfaces and in different environments.	➢ Largely depends on the user's skill and experience, which may introduce variability in treatment outcomes	➢ Print on demand in surgery	➢ Intensive training and introduction of standard operating procedures	[99,[103], [104], [105]]
Robotic arm-based printhead	➢ High degree of freedom for printing movement ➢ Possible for automated printing ➢ Possible for integration with imaging system ➢ Superior precision and repeatability	➢ Complex user interface and may pose injury risks ➢ High cost for manufacturing and maintaining	➢ Print on demand in surgery with pre-planned trajectories ➢ Possible for miniaturization to be used in internal endoscopic surgery	➢ Simplify the user interface and integrate additional safety sensors ➢ Reduce costs by introducing modular structures and standard parts	[100,101]

Importantly, functional printheads should be carefully selected for specific 3D bioprinting applications. For instance, printheads incorporating magnetic fields can accommodate a wider range of materials than acoustic-based printheads, which are constrained by material viscosity and acoustic properties. Material transport under magnetic fields is generally faster than under acoustic forces, enabling higher ink deposition rates. However, magnetic additives may negatively affect cell viability, whereas acoustic-based printheads can offer a more cell-friendly printing environment. Additional considerations are discussed in the relevant sections. It is also worth noting that multiple factors influence overall printing outcomes, including bioink formulation, printing parameters and operator expertise. All of these factors should be carefully evaluated and printing processes should be well planned to achieve optimal results.

To fully realize the potential of extrusion-based 3D printing, several challenges must be addressed. Compared with other additive manufacturing technologies, extrusion-based systems generally suffer from limited printing speed and resolution, primarily due to hardware and software constraints. Advances in hardware, such as dispensers with higher pressure-switching frequencies, faster and more precise motion stages and improved communication between auxiliary devices and the printer, could significantly accelerate the printing process. Resolution remains another critical limitation. Current strategies largely rely on reducing nozzle or needle diameters; however, this approach is often insufficient to meet the diverse requirements of multiple material printing, particularly when processing fragile, cell-laden bioinks. An alternative strategy to overcome these challenges is the integration of conventional molding techniques with 3D printing. While 3D printing enables high fidelity in reproducing fine structural details, molding can significantly accelerate fabrication and reduce cell damage associated with shear stress in conventional 3D bioprinting processes. For example, a kidney phantom was fabricated by first using wax-based 3D printing to create the inner collecting system, which was subsequently embedded in a mold and later removed after filling with mixtures containing hydrogels that mimic the appearance, imaging contrast and haptic properties of a human kidney [106].

Each of the innovative printhead technologies developed thus far presents unique trade-offs. Acoustic field-assisted printheads, for instance, require inks with embedded structures possessing specific geometric dimensions and acoustic contrasts, which limits material versatility. However, cells can be patterned in low viscosity media, which is promising. Magnetically assisted systems depend on inks with tailored magnetic properties, often demanding complex preparation steps that can impose stress on living components [107]. Similarly, electrically assisted printheads necessitate conductive or dielectric additives, raising concerns about cytotoxicity and extended preparation time. Furthermore, the use of magnetic additives can compromise cell viability, and the application of external electric fields (and to a lesser extent acoustic fields) can generate heat, which can alter ink rheology and compromise cell activity. Parallelization or integration of multi-module printheads will also be essential to achieve the throughput required for fabricating large-scale or clinically relevant constructs.

To facilitate the translation of these advanced printheads from academic laboratories to widespread adoption, standardized architectures, modular and interchangeable components, and robust calibration protocols will be essential. Especially for clinical applications, components that come into contact with sensitive biomaterials (e.g., nozzles and cartridges) should be disposable or easily sterilized. Different printhead designs offer distinct advantages, and many can be adapted for the fabrication of clinically relevant, human-scale tissues. Because cell-laden bioinks are highly sensitive, fabrication times should be constrained to the order of minutes. This requirement places increased importance on fabrication strategies when employing different 3D printing methods. In general, only critical regions with fine structural features should be fabricated using high-resolution printing, while other components can be produced with the largest feasible feature sizes to minimize overall fabrication time. As cellular constructs can fuse during culture [108], this modular printing approach also enables the separate printing of different regions of large, complex tissues followed by post-printing assembly. Such strategies further facilitate the incorporation of multiple cell types and biomaterials while reducing fabrication time through printing in parallel and the risk of contamination.

Looking forward, the integration of real-time monitoring and process optimization, which may be assisted by artificial intelligence (AI) tools, offers a promising route to overcome these limitations. Embedding feedback sensors within smart printheads could enable in situ monitoring of extruded strand fidelity, structural integrity and even cell viability. Coupled with adaptive parameter tuning from the control console, such systems would move extrusion printing towards true autonomous manufacturing. Additionally, for potential clinical applications of 3D bioprinting, it will be necessary to integrate real-time medical data analysis in the printing process to achieve adaptive printing and improve the speed and accuracy of model generation. This can enable more efficient bioprinting of tissues or organs for both medical training and therapeutic purposes [109]. 3D bioprinting will benefit from technological improvements in printing speed and resolution. Ultimately, these advances will expand the design space for multi-scale, multi-material structures across applications ranging from tissue engineering to soft robotics and intelligent material systems.

## CRediT authorship contribution statement

**Jianfeng Li:** Conceptualization, Data curation, Formal analysis, Investigation, Software, Visualization, Writing – original draft, Writing – review & editing. **Peer Fischer:** Conceptualization, Data curation, Formal analysis, Funding acquisition, Project administration, Supervision, Validation, Visualization, Writing – original draft, Writing – review & editing.

## Declaration of competing interest

The authors declare that they have no known competing financial interests or personal relationships that could have appeared to influence the work reported in this paper.

## Data Availability

Data will be made available on request.
